# c-CBL regulates melanoma proliferation, migration, invasion and the FAK-SRC-GRB2 nexus

**DOI:** 10.18632/oncotarget.10861

**Published:** 2016-07-27

**Authors:** Minakshi Nihal, Gary S. Wood

**Affiliations:** ^1^ Department of Dermatology, University of Wisconsin, School of Medicine and Public Health, Madison, Wisconsin, USA; ^2^ Paul P. Carbone Comprehensive Cancer Center, Madison, Wisconsin, USA; ^3^ Wm. S. Middleton VA Medical Centre, Madison, Wisconsin, USA

**Keywords:** c-CBL, melanoma, proliferation, migration, invasion

## Abstract

Melanoma is one of the most aggressive and lethal forms of skin cancer. Despite recent improvements in targeted therapies, many patients with advanced disease fail to achieve lasting tumor regression. Therefore, it is important to develop novel druggable targets that can be exploited to improve clinical outcome. Here, we studied the role of Casitas B-lineage lymphoma (c-CBL), an E3 ubiquitin ligase, in human melanoma. Employing quantitative real-time PCR and Western blot analysis in a panel of human melanoma cell lines (A375, G361, Hs-294T, SK-Mel-2, SK-Mel-28 and 451Lu), we found that c-CBL is strongly expressed in human melanoma cells at the mRNA and protein levels. Further, we determined c-CBL levels in clinical samples of melanomas and benign melanocytic nevi, using quantitative Nuance multispectral imaging. Compared to benign nevi, melanomas showed an overlapping range of c-CBL immunoreactivity. Small interfering RNA (siRNA)-mediated knockdown of c-CBL resulted in decreased proliferation, clonogenic survival and migration of melanoma cells. Furthermore, it also resulted in decreased cellular invasion in a 3D spheroid assay system. C-CBL and FAK are regulated by SRC, and FAK binds SRC and GRB2. C-CBL E3 ligase domain regulates receptor tyrosine kinase internalization through ubiquitination and its ring finger domain stabilizes the FAK-SRC-actin cytoskeleton thereby promoting cellular motility. C-CBL knockdown was associated with decreased protein and/or mRNA levels of SRC, FAK and GRB2. Taken together, we have provided evidence that c-CBL plays a role in melanoma cell proliferation, migration and invasion as well as inhibition of the FAK-GRB2-SRC nexus. Our findings indicate that additional studies are warranted to further dissect the role of c-CBL in melanoma and determine the therapeutic potential of its inhibition.

## INTRODUCTION

Melanoma being notoriously resistant to all available therapies has challenged the scientific community for several decades. Recent advances in the understanding of melanoma biology have resulted in some targeted therapies such as BRAF inhibitors for metastatic melanomas with BRAF-mutations. Also, the combination of BRAF and MEK inhibitors has improved progression-free survival, compared to standard monotherapy [1], and has received approval from the US FDA. However, even with combination therapies [2–5] patients develop acquired resistance and fail to achieve lasting tumor regression [6]. Therefore, intense research is ongoing to develop novel drugable targets, which could lead to the development of new approaches for melanoma management [7]. In this report, we studied the role of the ring finger ubiquitin E3 ligase, c-CBL, in melanoma.

The CBL proteins are a conserved family of ring finger ubiquitin ligases that have been shown to regulate signaling by tyrosine kinase (TK) receptors. The three known mammalian CBL proteins are encoded by separate genes: *c-CBL* (also known as *CBL2, NSLL, FRA11B, RNF55*), *CBL-b* (also known as *Nbla00127, RNF56*), and *CBL-c* (also known as *Cbl-3, Cbl-SL, RNF57*) [8]. The oncogenic v-c-CBL, from a dual-recombinant murine retrovirus that induces early B-lineage lymphomas, shares homology with both mouse and human forms of the ligase [9]. Mutations of c-CBL have been reported in a few myeloid and lymphoid neoplasms [10], and there is limited evidence suggesting that disruption of c-CBL function may contribute to the pathogenesis of other solid tumors as well [11–15]. Germline c-CBL mutations are associated with Noonan-like syndromes, sometimes referred to as “ras-opathies” [16].

C-CBL is a 120 kDa cytoplasmic protein that functions both as a multivalent adaptor and an E3 ubiquitin-protein ligase for many receptor protein tyrosine kinases and substrates [7, 17–19]. C-CBL is expressed in a wide range of tissues and cell types [20]. Defects in c-CBL proteins are suggested to be associated with malignancy and/or immune dysfunction [7]. The role of c-CBL in proliferative cell signaling and cancer has been suggested by a limited number of studies. We have recently shown that c-CBL is overexpressed in cutaneous T-cell lymphoma (CTCL) and modulates PLCγ-1-Fas apoptosis pathway signaling [21]. Kim *et al*. have shown that c-CBL shRNA-expressing adenovirus sensitized TRAIL-induced apoptosis in prostate cancer via increasing DR4/DR5 [22]. Met-dependent loss of CBL protein in MET-amplified gastric cancer cell lines represents another mechanism contributing to signal dysregulation in gastric cancer [23]. Shivanna and colleagues have shown that c-CBL ubiquitin ligase regulates nuclear β-catenin and angiogenesis by affecting its tyrosine phosphorylation via modulations in Wnt signaling, suggesting potential therapeutic benefit of targeting c-CBL in angiogenesis-associated diseases, including cancer [24, 25]. These studies provided us a rationale to determine the role of c-CBL in melanoma. We found that melanoma cells and lesional tissues exhibit c-CBL expression and its inhibition results in a decrease in proliferation, clonogenic survival, migration and invasion. These effects were found to be associated with reduced FAK-SRC-GRB2 transcript and protein levels.

## RESULTS

### C-CBL expression in human melanoma cells and tissue samples

**Figure 1:**
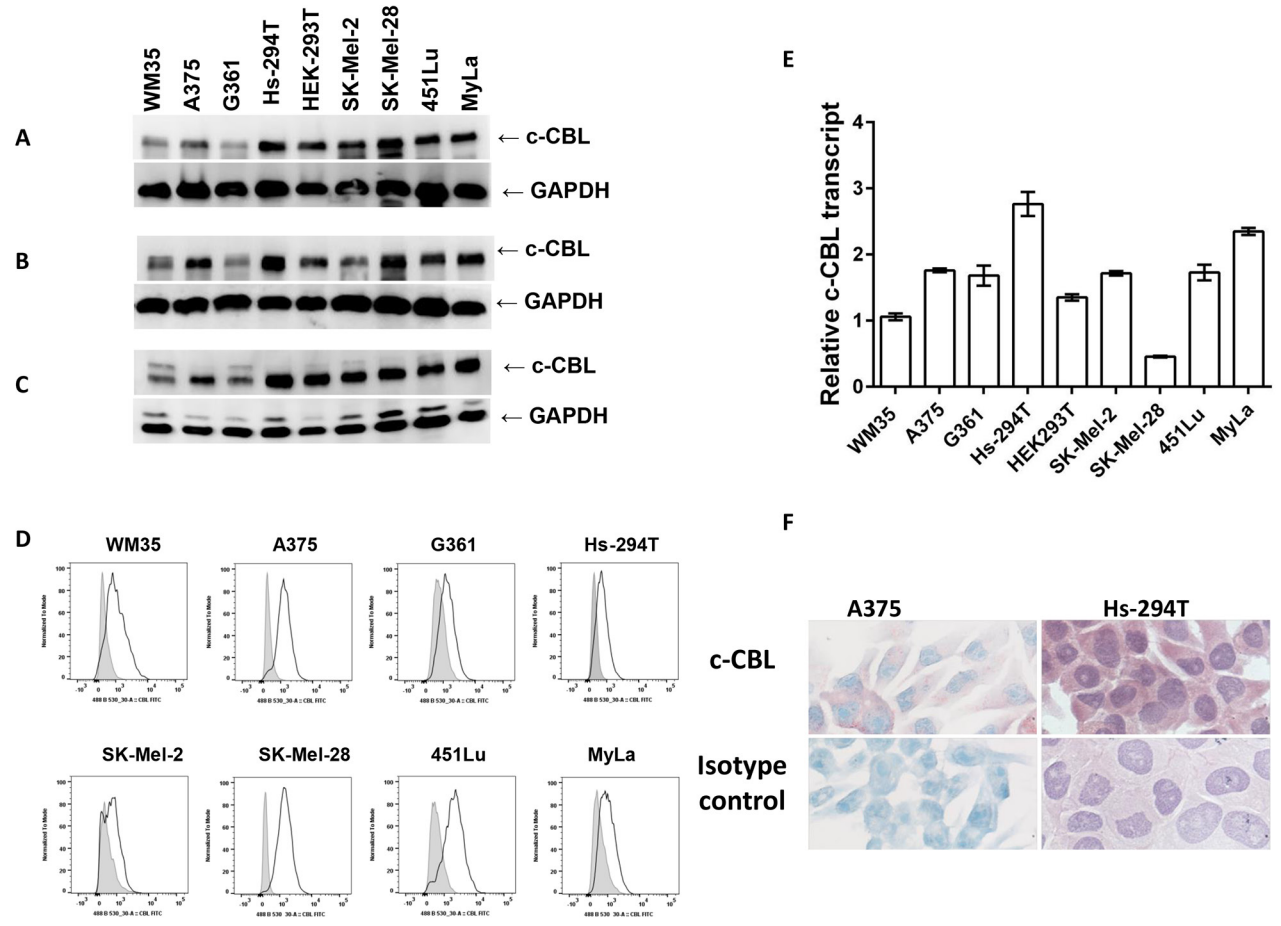
Melanoma cells exhibit c-CBL expression in cell lines Immunoblots showing c-CBL levels using three different monoclonal antibodies directed against different regions of the c-CBL molecule. **A.** D4E10; **B.** YE315; and **C.** 3B12 clones as stated in materials and methods. Level of GAPDH is used as a loading control and shown below the c-CBL bands. **D.** Flow cytometric histograms showing intracellular c-CBL staining. Filled grey histogram shows isotype control, and black lines show c-CBL staining. **E.** Histogram showing differential levels of c-CBL transcript via QRT-PCR in melanoma cell line panel. **F.** Intracellular staining of Warp red developed c-CBL in A375 counter stained with methylene blue at 90 X and Vulcan red stained Hs-294T counter stained with hematoxylin at 90 X. The immunoblots, flow cytometry, QRT-PCR experiments were done in triplicates and the results are presented as means ± SEM.

As shown in Figure 1, we checked the expression level of c-CBL in a panel of human melanoma cell lines using three different monoclonal antibodies reactive against different regions of the c-CBL protein (detailed in the method section). Clone D4E10 targets the Pro 582 region (Figure 1A); YE315 targets AA 480-520 (Figure 1B); 3B12 targets AA 684-865 (Figure 1C). CTCL MyLa cells were used as a positive control. In immunoblots, most of the melanoma lines showed prominent c-CBL expression with all the three antibodies. WM35 and G361 showed weaker expression with all three antibodies. SK-Mel-2 showed a bit weaker expression with antibody YE315. Next, we confirmed intracellular c-CBL immunoreactivity in melanoma cells via flow cytometry (Figure 1D). G361, Hs-294T and SK-Mel-2 were slight dimmer in c-CBL reactivity compared to other cells. Further, quantitative RT-PCR was performed to evaluate c-CBL transcript levels. We found WM35 and SK-Mel-28 showed a less transcript compared to other lines (Figure 1E). C-CBL immunoreactivity was also checked in melanoma cells grown in chamber slides. Figure 1F shows c-CBL immunoreactivity in AP Warp red developed A375 and AP Vulcan red developed Hs-294T cells.

**Figure 2:**
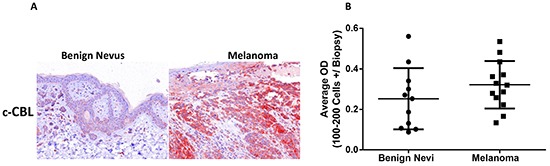
Melanoma cells exhibit c-CBL expression in human clinical samples C-CBL immunoreactivity in UV-APR stained and hematoxylin counterstained benign nevi and melanoma tissue samples as stated in materials and methods **A.** C-CBL immunostained benign nevi and melanoma tumor samples analyzed using multispectral imaging software (Nuance system) (Figure 2A). The histogram showing c-CBL immunoreactivity and each dot depicts the average OD value of 100-200 cells in each biopsy sample **B.**

In-situ c-CBL immunoreactivity was quantified in clinical melanoma and benign nevi tissue sections using the Nuance multispectral imaging system. Stained tissue images (Figure 2A) were digitized and measured for the amount of c-CBL signal intensity as optical density (OD) units. Clinical melanoma samples did not show statistically significant higher c-CBL immunoreactivity than benign nevi (Figure 2B). Each dot represents the average OD value of 100-200 cells in each biopsy sample as shown in histogram.

In summary, although there was some variation among the samples, c-CBL was found to be uniformly expressed by all melanoma cell lines and lesional tissue samples (Figures 1 and 2).

### C-CBL regulates melanoma cell proliferation, migration, invasion and spheroid formation

**Figure 3:**
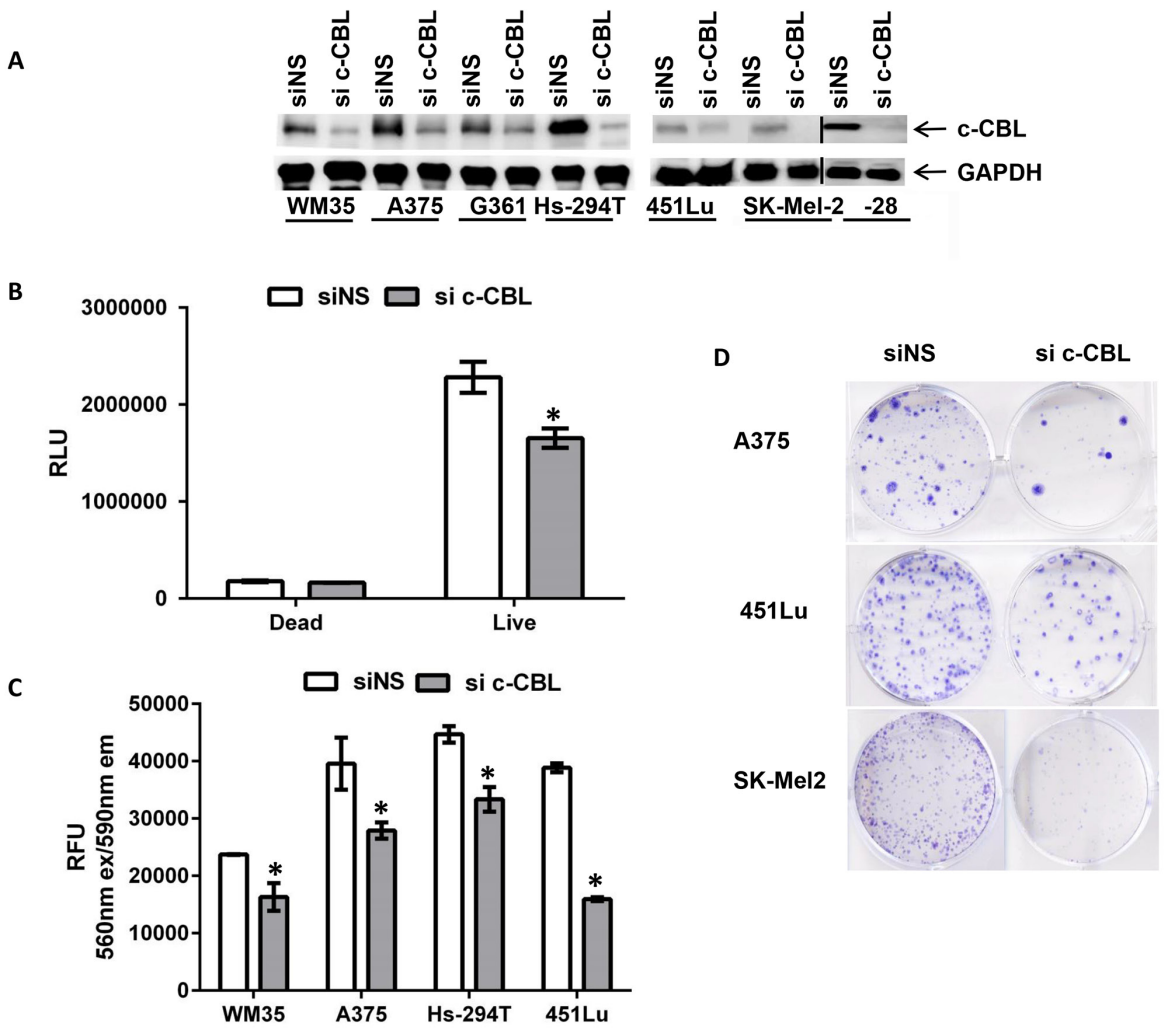
Knockdown of c-CBL reduces cellular viability and clonogenicity of melanoma cells **A.** Immunoblots showing c-CBL knockdown. GAPDH is used as a loading control and shown below. The vertical black line denotes junction with another immunoblot. **B.** Histogram showing reduced cellular growth when assayed with luminescent based Cytotox glow assay in A375 and **C.** with the Resorufin in control (siNS) and c-CBL knockdown (si c-CBL) cells. **D.** Multiple human melanoma cell line showing colonies stained with crystal violet in control (siNS) and c-CBL knockdown (si c-CBL) cells. All the experiments were done in triplicates and the results are presented as means ± SEM. Statistical significance is indicated as *P<0.05.

We knocked down c-CBL expression via electroporation of c-CBL siRNA (si c-CBL) into melanoma cells. The extent of the knock down was evaluated by immunoblot analysis as shown in Figure 3A. Contrary to our findings on c-CBL knock down in CTCL, down regulation of c-CBL in melanoma did not result in cellular death. Dead cell luminescence remained the same in both control and knockdown groups when assayed with a luminescent based CellTox glow assay in A375 cells (Figure 3B). We found that cellular growth was reduced in these cells with the same assay as shown in Figure 3B. Further, cellular growth in other c-CBL knock-down melanoma cell lines was measured with a non-fluorescent viability reagent, Resazurin, which is reduced by viabile cells to fluorescent Resorufin (ex530nm/em 590nm) in the mitochondria. C-CBL knock down caused reduced fluorescence in the mitochondria as a measure of reduced proliferation (Figure 3C). We also checked the clonogenic potential of melanoma cells with c-CBL knock down. Clonogenic output of melanoma cells with c-CBL knock down resulted in reduced colonies compared to nonsense control (Figure 3D).

Further, c-CBL knock down resulted in minimal apoptosis in A375 cells (annexin V/PI assay), and no change in Hs-294T cells (Supplementary Figure S1), showed no indication of senescence activity with β-galactosidase staining (the senescence marker) (Supplementary Figure S2), and caused no change in cell cycle distribution (Supplementary Figure S3).

**Figure 4:**
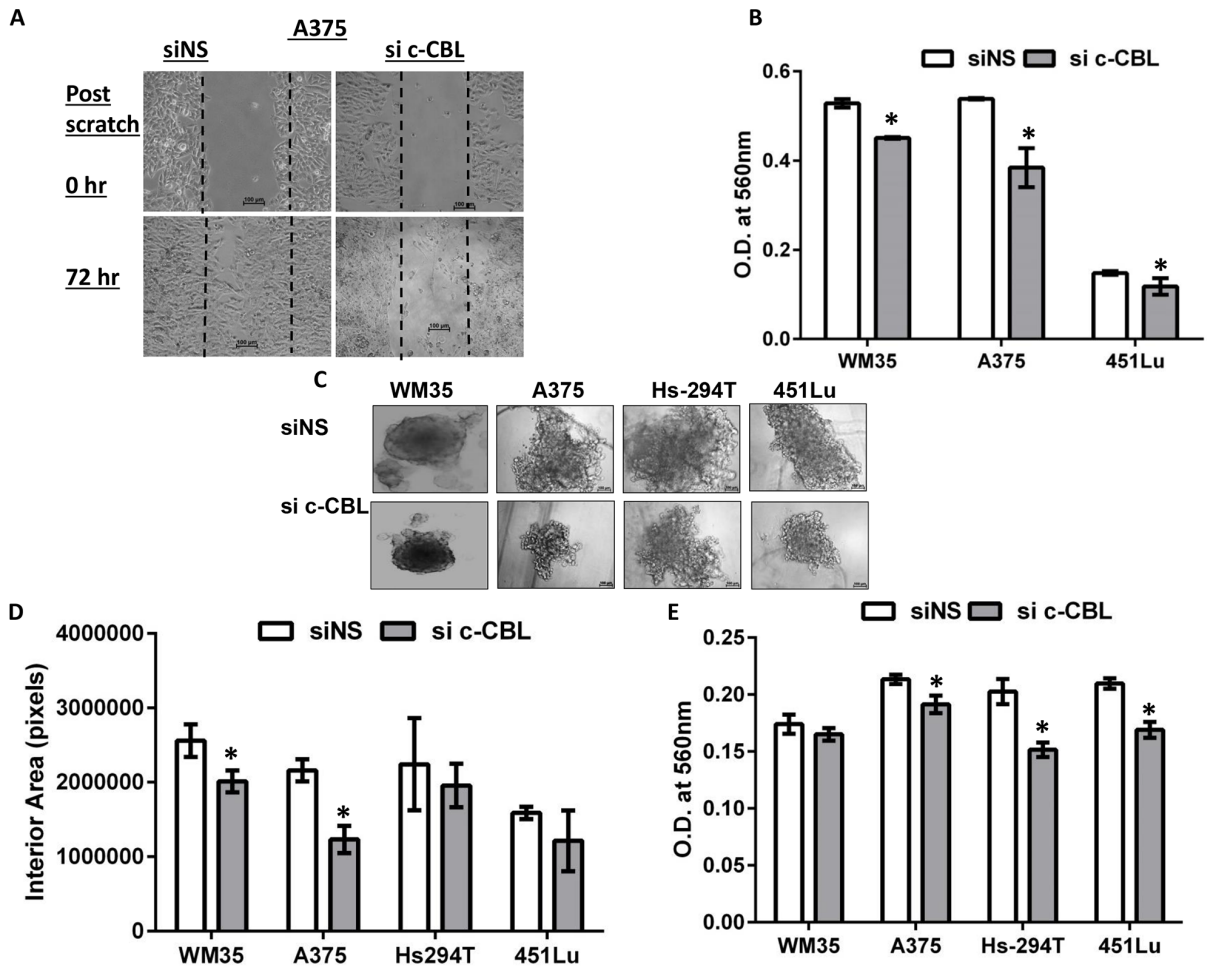
Knockdown of c-CBL reduces migration, invasion and 3D spheroid proliferation and invasion **A.** Scratch (wound) assay in A375 cells showing reduced migration in in c-CBL knock down A375 cells. **B.** Trans- well migration of the cells across polycarbonate microporous membrane inserts is shown as the histogram. The Y-axis represents the OD of migrated control and c-CBL knockdown cells (at the bottom of membrane) at 560 nm. **C.** Colony formation ability of control and c-CBL knockdown melanoma cells on extracellular matrix (ECM) showing representative images after 7 days in culture. **D.** Images of the colonies were photographed and the digitized images were measured for interior area of spheroids and plotted as a histogram. **E.** At the conclusion of the assay, cellular metabolic activity was assessed using MTT reagent and plotted as a histogram. **F.** Histograms showing measurement (as in C) of a 3D culture invasion assay of control and c-CBL knockdown melanoma cells across a special BME basement membrane extracellular matrix over a 50- day period. All the experiments were done in triplicates and the results are presented as means ± SEM. Statistical significance is indicated as *P<0.05.

Next we determined whether c-CBL affects the migration of melanoma cells. After c-CBL knockdown A375 cells showed reduced migration with the scratch wound assay (Figure 4A), which is a measure of basic cell migration parameters such as speed, persistence, and polarity. We also checked cellular invasion through microporous membrane inserts. C-CBL knock down cells exhibited reduced trans-well invasion across inserts shown as a histogram in Figure 4B. The clonogenic spheroid formation ability of melanoma cells was also tested. Figure 4C shows that the spheroids formed with c-CBL knock down cells were smaller than those formed by controls. The interior areas of digitized spheroid images were plotted as a histogram (Figure 4D). C-CBL knock down spheroids exhibited reduced metabolic activity as documented using the end point MTT assay (Figure 4E). Further, a 3D invasion through a basement membrane-like barrier, consisting of extracellular matrix and collagen (BME), was also performed to determine the tumor invading potential of these cells. Invasion was visualized microscopically and images were procured and quantified similar to Figure 4D over a period of 50 days and represented as a histograms (Figure 4F). The data showed a uniform downward trend in invasion among all cell lines with statistical significance achieved in various cell lines in the 10-50 day range.

### C-CBL knockdown affects the FAK-SRC-GRB2 nexus

**Figure 5:**
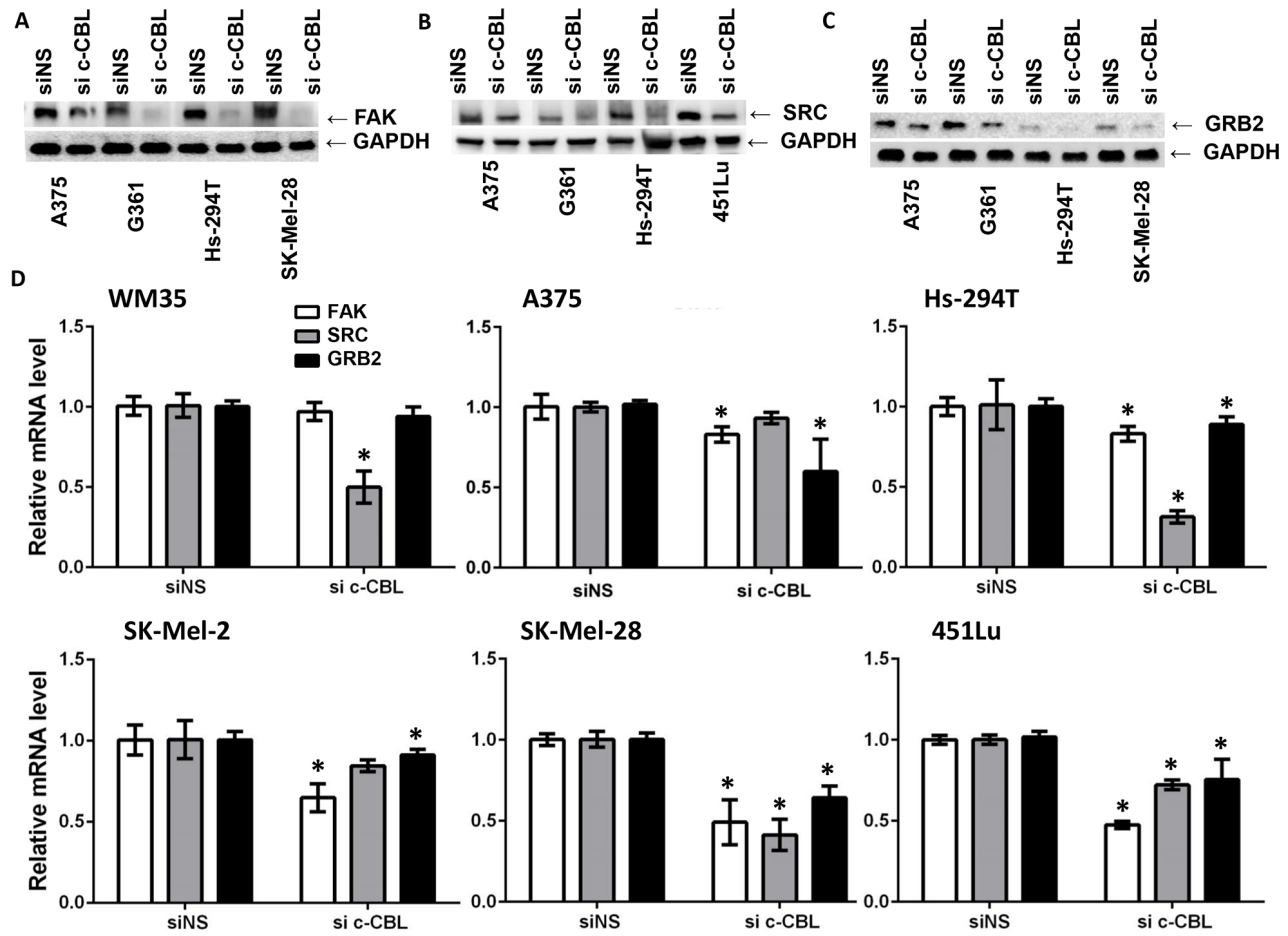
C-CBL knockdown inhibits the FAK-GRB2-SRC nexus Knockdown of c-CBL decreased FAK **A.**, SRC **B.** and GRB2 **C.** protein expression as shown in immunoblots. GAPDH is used as a loading control and shown below. C-CBL knockdown reduced FAK, GRB2 and SRC mRNA **D.** All the experiments were done in triplicate and the results are presented as means ± SEM. Statistical significance is indicated as *P<0.05.

C-CBL is regulated by non-receptor tyrosine kinases SRC (v-src sarcoma (Schmidt-Ruppin A-2) viral oncogene homolog (avian)), which is upstream of FAK (focal adhesion kinase, also known as PTK2). FAK, binds to SRC and GRB2 (growth factor receptor bound protein 2), and is also regulated by SRC. It has a known role in cellular proliferation, migration, apoptosis, adhesion and growth as well as a plethora of other biological processes [26, 27]. Therefore, we hypothesized that alterations of the FAK-SRC-GRB2 nexus might be associated with the effects of c-CBL knockdown on the potential of melanoma cells to proliferate, migrate and invade. We found that c-CBL knockdown resulted in reduced FAK (Figure 5A), SRC (Figure 5B) and GRB2 (Figure 5C) protein expression in several melanoma cell lines. Transcripts of FAK, SRC and GRB2 were also found significantly decreased in several lines (Figure 5D).

## DISCUSSION

In this study of human melanoma, we determined the expression and functional consequences of inhibiting the proto-oncogenic E3 ubiquitin ligase, c-CBL. This is important because melanoma is known for its pro-proliferative signaling through multiple pathways, including Ras/Raf/MEK/ERK mitogen activated protein kinase (MAPK). Melanoma cells rapidly acquire resistance to targeted therapies, requiring drug combinations, which often result in only temporary clinical benefit. Thus, it is critical to identify novel molecular targets that can be exploited for the treatment of melanoma as either monotherapy or combination therapy.

C-CBL has been shown to have diverse roles as a regulator of signal transduction and an adaptor protein, and its dysfunction has been implicated in certain malignancies. In this study, we found that melanoma cell lines showed strong expression of c-CBL at both the mRNA and protein levels (Figure 1) and strong protein expression was also present in human melanoma tissue (Figure 2). Wild type c-CBL is reported as a tumor suppressor in several cancers and acts as a negative regulator of receptor and non-receptor protein tyrosine kinases via their degradation by polyubiquitination. C-CBL and its transforming mutants have displayed both negative and positive regulatory activities in protein tyrosine kinase and RAS/PI3K/AKT signaling [7]. C-CBL mutations have also been reported in a small percentage of acute lymphoblastic and monocytic leukemias. Only about 3 % of melanomas have shown c-CBL mutations as per TCGA data base [15]. Consistent with our results in melanoma, c-CBL protein has been shown to be overexpressed in prostate [22], gastric [23], pancreas [28], lung [12, 29, 30], primary colorectal cancers [14], glioma [13], myeloid [8, 10], acute lymphoblastic leukemia [31], chronic lymphocytic leukemia [32], and cutaneous T-cell lymphoma [21].

To determine the functional significance of c-CBL in melanoma, we performed knock-down of c-CBL using siRNA. This resulted in decreased proliferation, migration, colony formation and invasion of melanoma cells (Figure 3 and 4). Our findings are consistent with several published studies which have suggested a role for c-CBL in cytoskeletal events, such as cell spreading, adhesion, and migration. In prostate cancer, c-CBL was shown to regulate tumor cell adhesion, migration, and degradation, and to act as an oncogenic prognostic marker correlating with poor clinical outcome. Recently, Lee et al. have shown that wild type c-CBL expression by glioma cells promotes invasion through upregulation of MMP2 [13]. Because increased c-CBL blocks kinases that lead to activation induced cell death in cutaneous T-cell lymphoma cells, its knockdown promotes apoptosis of this neoplasm [21]. On the other hand, c-CBL appears to act as a tumor suppressor in other malignancies. A recent study on human pancreatic ductal adenocarcinomas validated that low c-CBL was associated with shorter survival, and had an inverse correlation with levels of epidermal growth factor receptor [28]. Ectopic c-CBL decreased the receptor tyrosine kinase levels in osteosarcomas and reduced tumor growth and metastasis by inhibiting cell proliferation, migration and invasion [33]. The loss of c-CBL function in individuals with leukemia and myelodysplastic syndrome supports the potential therapeutic value of wild type c-CBL therapy in some hematologic malignancies, in addition to those with lung cancer [34]. Thus, c-CBL seems to have a dichotomous role, acting both as a tumor suppressor or oncogene depending on the cancer type and its dominant pathogenic mechanisms [35, 36].

Our data demonstrate that the effects of c-CBL knockdown in melanoma (Figure 3), were accompanied by decreases in the protein and mRNA levels of FAK, SRC and GRB2 (Figure 5), suggesting the involvement of the FAK-GRB2-SRC nexus in the consequences of c-CBL alteration. This is significant because of the known interactions among these proteins and their functional consequences. Cell migration and invasion through FAK and SRC signaling pathway is implicated in focal adhesion turn-over in melanoma. C-CBL is a substrate for SRC- and SYK-family kinases, and is physically associated with them. FAK and SRC-dependent recruitment of c-CBL has been implicated in the regulation of cell attachment and motility [37]. FAK signaling has been shown to regulate proteins (e.g. SRC, SYK, FYN, LYN, GRB2, PI3K, and paxillin) that are involved in modulating cytoskeleton rearrangements to enhance tumor growth and metastasis [36, 38, 39]. FAK is overexpressed and activated in primary or metastatic cancers and has prognostic significance. FAK and SRC signaling also promote angiogenesis and protease-associated tumor metastasis [27]. In osteoclasts FAK is autophosphorylated upon cell attachment, and subsequently forms a complex with activated SRC and c-CBL [40, 41], [37, 42, 43]. The adaptor protein GRB2 also interacts with c-CBL and FAK [37, 44, 45], and plays an important role in Ras signaling pathways [46].

In summary, we have shown that c-CBL plays a supportive role in the proliferation, migration and invasion of human melanoma cells. Furthermore, c-CBL knockdown down-regulates the FAK-GRB2-SRC nexus, a system known to promote cell growth, proliferation and mobility of normal and neoplastic cells. Based on our novel findings, additional studies are warranted to further delineate the function of c-CBL in melanoma and to determine whether targeting it will have therapeutic benefit.

## MATERIALS AND METHODS

### Cell lines and siRNA treatments

Melanoma cells were obtained from The American Type Culture Collection (ATCC; VA) and were maintained at standard tissue culture conditions as recommended by the vendor. Control (1027280) and c-CBL (1027416) siRNA were obtained from Qiagen and used at a final concentration of 30 nm via electroporation using Amaxa cell line Nucleofactor kit V and program X-001.

### Immunoblot analysis

Untreated, siNS and si c-CBL transfected cell pellets were washed with ice-cold 1X PBS and lysed with 1X RIPA buffer. Protein samples (30-50 μg) were subjected to SDS-PAGE and transferred onto nitrocellulose membrane. Blots were exposed to anti-c-CBL antibodies [{Cell signaling (D4E10); directed against the region around Pro 582}, {LS Bio (YE315) directed against central; AA 480-520 (P22681)}, and {Thermo Fisher (3B12) directed against AA 684-865}], anti-FAK (3285), anti-SRC (2123), anti-GRB2 (3972), and loading control anti-GAPDH (2118) primary antibodies (1:100) and HRP conjugated appropriate secondary antibodies (all from Cell Signaling, Inc., Danvers, MA), followed by enhanced chemiluminescent detection (Thermo Fisher Scientific, Inc., IL). Represented blots are from three independent experiments with similar results.

### Flow cytometry

Untreated, siNS and si c-CBL melanoma cells (1×10^5^) were collected, washed with PBS twice and fixed with 4 % formaldehyde for 10 min at room temp, followed by cell permeabilization with 1 % Triton-X100 and again twice washed with PBS then blocked with 10 % NGS for 15 min at room temperature and incubated with anti-c-CBL antibody (TF# MA5-15885; 3B12) at 1:125 dilution for 4 h at 4°C. Goat-anti-mouse FITC antibody (BD # 555988) (1:50) was used as secondary antibody for 30 min in dark. Cells were washed with PBS with 0.5 % BSA twice and subjected to flow cytometry. For isotype control 20 μl of the FITC mouse IgG1k was used (BD# 555748). After staining, flow cytometric analysis was performed with a LSRII (BD Biosciences, CA) at the UWCCC Flow Cytometry Facility using FlowJo software (Treestar, OR).

### Quantitative real time PCR (QRT-PCR)

For QRT-PCR, RNA was isolated with Trizol reagent (Invitrogen, CA), treated with DNAse (Promega, WI) and first strand cDNA created with M-MLV reverse transcriptase (Promega, WI) according to vendor's protocol. QRT-PCR was performed, in triplicate, with SYBR Premix Ex Taq Perfect Real Time (Takara, WI) with first strand cDNA for FAK (PrimerBank ID 313851041c1), SRC (PrimerBank ID 38202216c1), GRB2 (PrimerBank ID 156071491c1) and GAPDH (PrimerBank ID 126273608c1) [47]. Relative expression of the transcript was calculated using the ΔΔCT method using GAPDH as an endogenous control.

### Immunocytochemical staining (ICC)

C-CBL immunoreactivity was assessed via immunocytochemical analysis. Cells were grown in chamber slides and fixed with 4 % methanol free paraformaldehyde solution followed by PBS wash and then permeabilized with 0.2 % Triton X-100 in PBS for 10 min. Slides were incubated with anti c-CBL (LSBio clone YE315; at 1:100 dilution) overnight at 4 °C. A375 cells were stained with Warp red labeled alkaline phosphatase and counter stained with methylene blue. Hs-294T cells were stained with labeled alkaline phosphatase Vulcan red stained and counter stained with hematoxylin. Cover slips were mounted with permount solution. Images were captured with Nikon Digital Sight DS-Fi1 inverted microscope (Nikon Instruments Inc., NY) using NIS Elements AR 3.1 software at 90 X magnification.

### UltraView universal alkaline phosphatase red (UV-APR) IHC staining and quantification

Tissue sections (5 μm thick) were de-paraffinized and subjected to heat induced antigen retrieval and then incubated with anti c-CBL (LSBio clone YE315; at 1:100 dilution) overnight at 4 °C. After 3 washes in PBS, sections were subjected to UVAPR detection kit which utilizes a cocktail of enzyme labeled secondary antibodies and then the complex is visualized with naphthol and fast red chromogen, which produces a red precipitate that is readily detected by light microscopy. Briefly, sections were incubated with 1-2 drops UVAPR universal multimer for 12 min, followed by 3 washes with PBS for 2 min each, universal multimer enhancer for 4 min, a drop each of UV fast red A and UV red naphthol for 8 min, and then a drop of UV fast red B; for 8 min, followed by counterstaining with hematoxylin for 30 sec and mounting. Staining controls included normal serum only, isotype antibody only, as well as counterstain only and immunostain only.

### In-situ multispectral image quantification

In-situ c-CBL expression was quantified using the Nuance multispectral imaging system (Perkin-Elmer, MA). This system uses a light microscope equipped with a liquid crystal tunable filter, high-resolution digital camera and a computer loaded with proprietary image analysis software (INFORM). The single color controls allowed acquisition of a multispectral signature for each chromogen in the staining system (here blue for hematoxylin II and red for c-CBL detected by UVAPR). Stained tissue sections were digitized and tumor cells of interest were circled individually within the image and the system measured the amount of UVAPR red signal intensity, automatically converting these data into optical density (OD) units. Positive staining was adjusted by subtracting background control signals. The results were recorded on a cell-by-cell basis as average OD value/cell and expressed as mean staining intensity on plots of individual tissue specimens. Expression of c-CBL was also shown in photomicrographs of immunostained tissues [47].

### Cell growth and viability assay

Cell growth and viability was assessed using the CytoTox-Glo assay system (Promega, WI) according to manufacturer's protocol. Briefly, 1×10^4^ siNS and si- c-CBL cells in a total of 100 μl medium were plated in a 96-well plate (n=6) for 48 h. Next, cells were incubated for 15 min with AAF-Glo substrate (alanyl-alanylphenylalanyl-aminoluciferin) that measures a distinct intracellular protease activity associated with cytotoxicity (dead-cell protease) via a luminescent signal. Cell viability was calculated by subtracting the luminescent signal of experimental cell death from total luminescent signal of total lysed cell. Data were represented as percent change in viable and dead cells. Since c-CBL knockdown did not induce much apoptosis, we checked the viability of rest of the cell lines and among both the groups using Resazurin (also known as Alamar blue) which offers a simple, rapid and sensitive measurement for the viability of mammalian cells. Plating density, format and end point for evaluation were all same as above. Metabolically active living cells are able to reduce the non-fluorescent dye Resazurin to the strongly-fluorescent dye Resorufin. The fluorescence output was proportional to the number of viable cells. Data were represented as percent reduction in viable cells normalized to siNS control (n=3).

### Clonogenic cell survival assay

Control and si c-CBL cells were seeded at a density of 3 × 10^3^ in 6-well plates. After 14 days, colonies were fixed with 6% glutaraldehyde, washed twice, and stained with 0.5 % crystal violet in acetic acid (methanol: H2O: acetic acid 1:2:2) (Sigma- Aldrich, MO). Stained colonies were washed twice with 1X PBS, and photographed.

### Wound healing/scratch assay

Cell migration among control and si c-CBL cells was examined using a wound-healing assay. In brief, 0.5×10^6^ cells were placed in a well of six-well plates and at confluence a scratch/wound was made with a 10 μl pipette tip followed by washing with serum-free medium to remove cell debris. Wells were photographed under phase-contrast microscopy (time=0) and then cells were allowed to migrate into the scratch/wound area for up to 72 h at 37°C and photographed under Nikon Digital Sight DS-Fi1 camera using NIS Elements AR 3.1 software.

### Chemotaxis assay

Trans-well migration was assessed using Millipore's 24-well plate and 8 μm pore size polycarbonate membrane inserts. After plating the control and c-CBL knock down cells for 24 h in the migration chambers, cells that have migrated through the polycarbonate membrane at the bottom of membranes were dislodged, and collected. Cells that have migrated through the polycarbonate membrane were incubated with cell stain solution, then subsequently extracted and detected on a microplate reader at 560 nm. Data were represented as percent reduction in migratory cells normalized to no treatment control.

### 3D Colorimetric spheroid proliferation assay

Cell proliferation in a 3D culture was investigated as per manufacture's protocol (Trevigen, Inc). Spheroid formation extracellular matrix (ECM) was thawed on ice for two hours and 3×10^3^ cells were mixed with 50 μl ECM (1:10 with culture medium) in the cold and centrifuged at 200 × *g* for 3 min at room temperature in a swinging bucket rotor, then Incubated at 37°C in a humidified incubator for 72 h to promote spheroid formation. Spheroids in each well were photographed every 24 h using the 4 X objective. At the end of 7 days 10 μl of MTT reagent was added per well and the plates were incubated at 37°C for 24 h. An equivalent volume of detergent reagent was added per well and the plates were incubated at 37°C incubator for additional 24 h. Absorbance was read as OD at 570 nm and the histogram shows the difference in the metabolic activity of control and si c-CBL cells.

### 3D Spheroid BME cell invasion assay

Cell invasion in a 3D Culture used the spheroid formation protocol as stated above. Then thawed basement membrane extract (BME), derived from murine EHS sarcoma cells and collagen invasion matrix, along with the spheroid formation plate was placed on ice for 15 min. Working on ice, 50 μl of BME was added per well and the plate was centrifuged at 300 × *g* at 4°C for 5 min in a swinging bucket rotor to eliminate bubbles and position spheroids within the invasion matrix towards the middle of the well. Plates were then incubated at 37°C in a humidified incubator for 50 days, and the spheroid in each well was photographed every 5th day using the 4 X objective.

### Statistical analysis

Statistical analyses for all the experiments were performed with two-tailed unpaired Student's t-test between two experimental groups using PRISM version 5.0 software (GraphPad Software, Inc., CA). Data were expressed as mean ± SEM unless otherwise stated. P value ≤ 0.05 was considered significant.

## SUPPLEMENTARY MATERIAL METHODS AND FIGURES


