# Supplementary material for: c-CBL regulates melanoma proliferation, migration, invasion and the FAK-SRC-GRB2 nexus
Source: Oncotarget. 2016 Jul 27;7(33):53869–80. doi: 10.18632/oncotarget.10861 (PMC5288227; doi:10.18632/oncotarget.10861)
Supplement: Supplementary file 1 [file oncotarget-07-53869-s001.pdf]

c-CBL regulates melanoma proliferation, migration, invasion and the FAK-SRC-GRB2 nexus

SUPPLEMENTARY METHODS AND FIGURES

Annexin V and PI staining for apoptosis

Cells apoptosis was measured with Annexin v binding. In brief, control (siNS) and si c-CBL melanoma cells were seeded in 12-well plates at a density of 1×10^5 cells/ml with fresh complete culture medium for 48 h. Afterwards, cells were trypsinized and resuspended in Annexin V binding buffer, Annexin V–FITC antibody (5 µl) and PI (5 µl), vortex-mixed gently and incubated for 15 min at room temperature in the dark. Cells were analyzed by flow cytometer (Becton–Dickinson, MA) and FlowJo software.

Senescence-associated beta-galactosidase (SA-β-Gal) staining

Control (non sense) and c-CBL knockdown melanoma cells were seeded at a density of 5.0×10^5 in 6-well plates. After being washed and fixed for 5 min at room temperature in 6 % glutaraldehyde, they were incubated overnight at 37°C (without CO2) with freshly prepared SA- β-gal stain solution (1 mg/ml X-gal, 40 mM citric acid/ sodium phosphate (pH 6.0), 5 mM potassium ferrocyanide, 5 mM potassium ferricyanide, 150 mM NaCl, 2 mM MgCl2) (blue color sedimentation in their cytoplasm). Afterwards wells were washed with PBS, and nuclei were counterstained with nuclear fast red stain. Images were captured under the Nikon Digital Sight DS-Fi1 camera using NIS Elements AR 3.1 software.

Cell cycle analysis

Control and c-CBL knockdown cells were seeded at a density of 1×10^5 in 6-well plates and collected after 48 h, washed with PBS, detached with 0.25 % trypsin and fixed with 70 % ethanol. Cells were treated with 1 mg/ml RNase A (Sigma- Aldrich, MO) at 37°C for 30 min, and resuspended in 0.25 ml of PBS and stained with propidium iodide (PI) (Sigma- Aldrich, MO) (final conc 50 µg/ml.). Stained cells were analyzed using a FACScan flow cytometer (Becton–Dickinson, MA) and cell cycle distribution was analyzed using ModFit software (Verity Software, ME) and shown as a histogram.

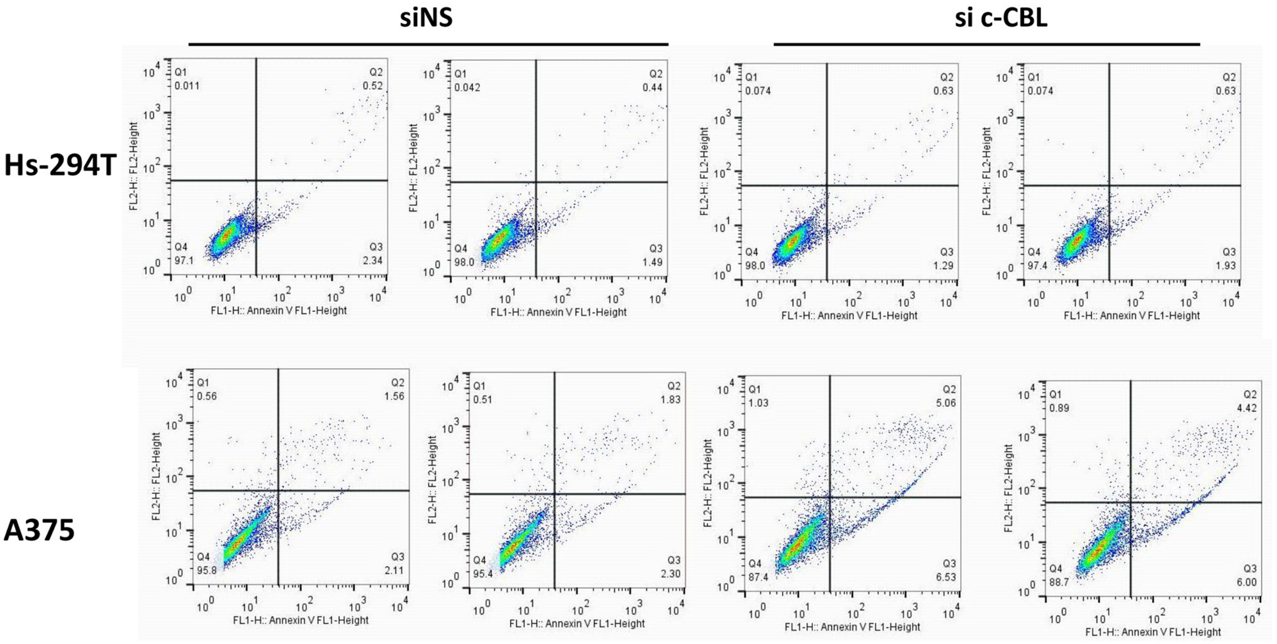

Supplementary Figure S1: Dot plot of Annexin V/ propidium iodide stained apoptosis assay showing no effects of c-CBL knockdown in Hs-294T and a minimal effects in A375 human melanoma cells.

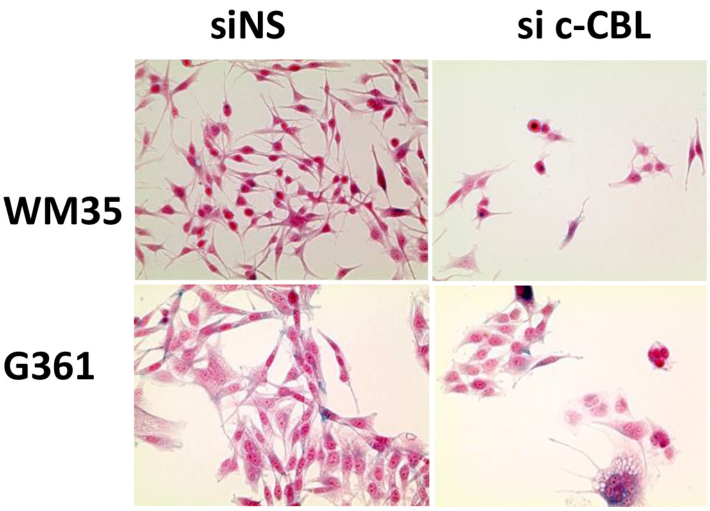

Supplementary Figure S2: Control and c-CBL knockdown cells stained for β-galactosidase senescence activity, counter stained with nuclear fast red. Stained cells showed no senescent activity with c-CBL knockdown.

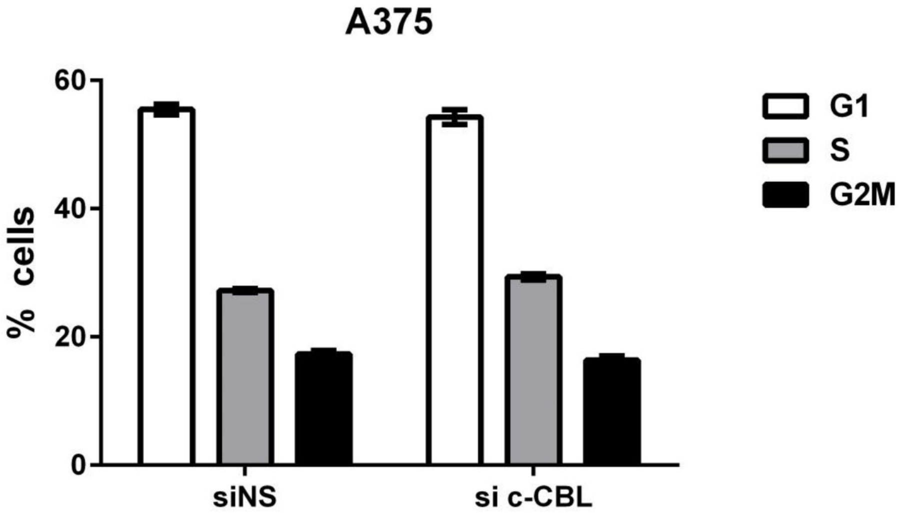

Supplementary Figure S3: Cell cycle phase distribution among control and c-CBL knockdown A375 human melanoma cells.
